# AGOUTI: improving genome assembly and annotation using transcriptome data

**DOI:** 10.1186/s13742-016-0136-3

**Published:** 2016-07-19

**Authors:** Simo V. Zhang, Luting Zhuo, Matthew W. Hahn

**Affiliations:** School of Informatics and Computing, Indiana University, Bloomington, IN 47405 USA; Department of Biology, Indiana University, Bloomington, IN 47405 USA

**Keywords:** Genome assembly, Scaffolding, Genome annotation, RNA sequencing, RNA-seq

## Abstract

**Background:**

Genomes sequenced using short-read, next-generation sequencing technologies can have many errors and may be fragmented into thousands of small contigs. These incomplete and fragmented assemblies lead to errors in gene identification, such that single genes spread across multiple contigs are annotated as separate gene models. Such biases can confound inferences about the number and identity of genes within species, as well as gene gain and loss between species.

**Results:**

We present AGOUTI (Annotated Genome Optimization Using Transcriptome Information), a tool that uses RNA sequencing data to simultaneously combine contigs into scaffolds and fragmented gene models into single models. We show that AGOUTI improves both the contiguity of genome assemblies and the accuracy of gene annotation, providing updated versions of each as output. Running AGOUTI on both simulated and real datasets, we show that it is highly accurate and that it achieves greater accuracy and contiguity when compared with other existing methods.

**Conclusion:**

AGOUTI is a powerful and effective scaffolder and, unlike most scaffolders, is expected to be more effective in larger genomes because of the commensurate increase in intron length. AGOUTI is able to scaffold thousands of contigs while simultaneously reducing the number of gene models by hundreds or thousands. The software is available free of charge under the MIT license.

**Electronic supplementary material:**

The online version of this article (doi:10.1186/s13742-016-0136-3) contains supplementary material, which is available to authorized users.

## Background

### Findings

Genomes sequenced using short-read, next-generation sequencing technologies are fragmented into hundreds, sometimes even thousands, of small sequences [1]. In addition to a general lack of data about sequence contiguity, one consequence of fragmented genome assemblies is that single genes are placed on multiple contigs or scaffolds, increasing the number of predicted genes [2]. Such biases can confound inferences about the number and identity of genes within species, as well as gene gain and loss between species [3].

Data from expressed genes, that is, transcriptome or RNA sequencing (RNA-seq) data, has previously been used to combine contigs into scaffolds (e.g., [4, 5]), acting in effect as a mate-pair library with insert size equivalent to intron length. Such approaches have been shown to be able to improve genome assembly by increasing contiguity [6]. However, they do not generally decrease the number of incorrectly predicted genes. This is because contigs within scaffolds are connected by gaps, and gene prediction programs cannot predict across gaps of even moderate length. However, we previously showed that RNA-seq can also be used to reduce the number of gene models split apart by fragmented assemblies because it contains information about connections between exons in a single gene [2].

Here we combine these two uses of transcriptome data into a single lightweight program that we call AGOUTI (Annotated Genome Optimization Using Transcriptome Information). As with other scaffolders based on RNA-seq, AGOUTI brings together contigs into scaffolds, yielding a more contiguous assembly. It does this with an algorithm similar to the one used in RNAPATH [5], but with additional denoising steps and constraints that ensure greater accuracy. AGOUTI also simultaneously updates gene annotations by connecting predictions from multiple contigs, significantly reducing the number of gene models initially predicted from draft assemblies. We are not aware of other annotation software that has these features.

### Algorithm

**Fig. 1:**
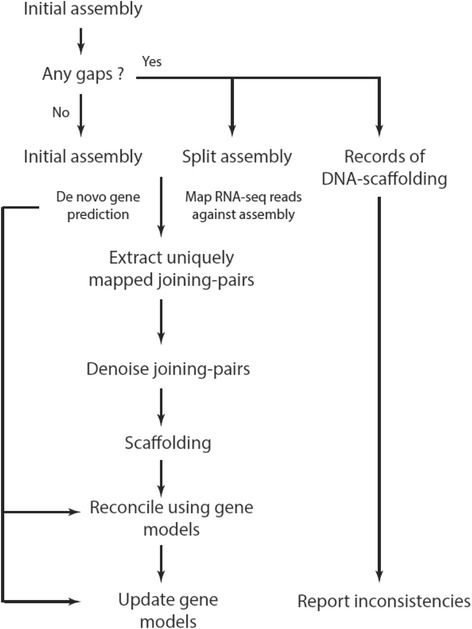
AGOUTI workflow

An overview of AGOUTI is given in Fig. 1. The method takes three inputs: an initial genome assembly in FASTA format, paired-end RNA-seq reads mapped against this assembly in BAM format, and gene predictions from the initial assembly in GFF format. The output of AGOUTI is an updated genome assembly file (in FASTA format) and an updated set of gene predictions (in GFF format). AGOUTI accepts assemblies as both contigs and scaffolds. In scaffold form, AGOUTI optionally breaks assemblies at gaps of certain lengths, essentially reducing them to contig form (a ‘split’ assembly). AGOUTI scaffolds on split assemblies and will report inconsistencies between the RNA-based scaffolding it conducts and the original scaffolding. These inconsistencies can also provide valuable evidence of errors in the original assembly [6, 7].

### Extracting joining-pairs

AGOUTI starts by identifying ‘joining-pairs’, pairs of reads that are mapped to different contigs. It is through these pairs that many of the existing scaffolding algorithms are able to assemble contigs into scaffolds (e.g., [5–9]). AGOUTI uses only those joining-pairs that are uniquely mapped, recording the mapping positions and orientations for all identified pairs. Short-read mappers such as BWA-MEM [10] and Bowtie2 [11] use a non-zero mapping quality to determine the uniqueness of an alignment. Besides mapping quality, AGOUTI provides two additional parameters accessible from the command line to filter out suspicious alignments: maximum percentage of mismatches per alignment allowed (-maxFracMM; 5 % by default), and minimum percentage of alignment length allowed (i.e., the ratio of the alignment length to the read length; -minFracOvl; 70 % by default). Each filter is applied to both ends of a pair. These two options can be disabled by specifying 100 % mismatch rate and 0 % alignment length. All of our AGOUTI evaluations were conducted with these two parameters disabled.

### Denoising joining-pairs

**Fig. 2:**
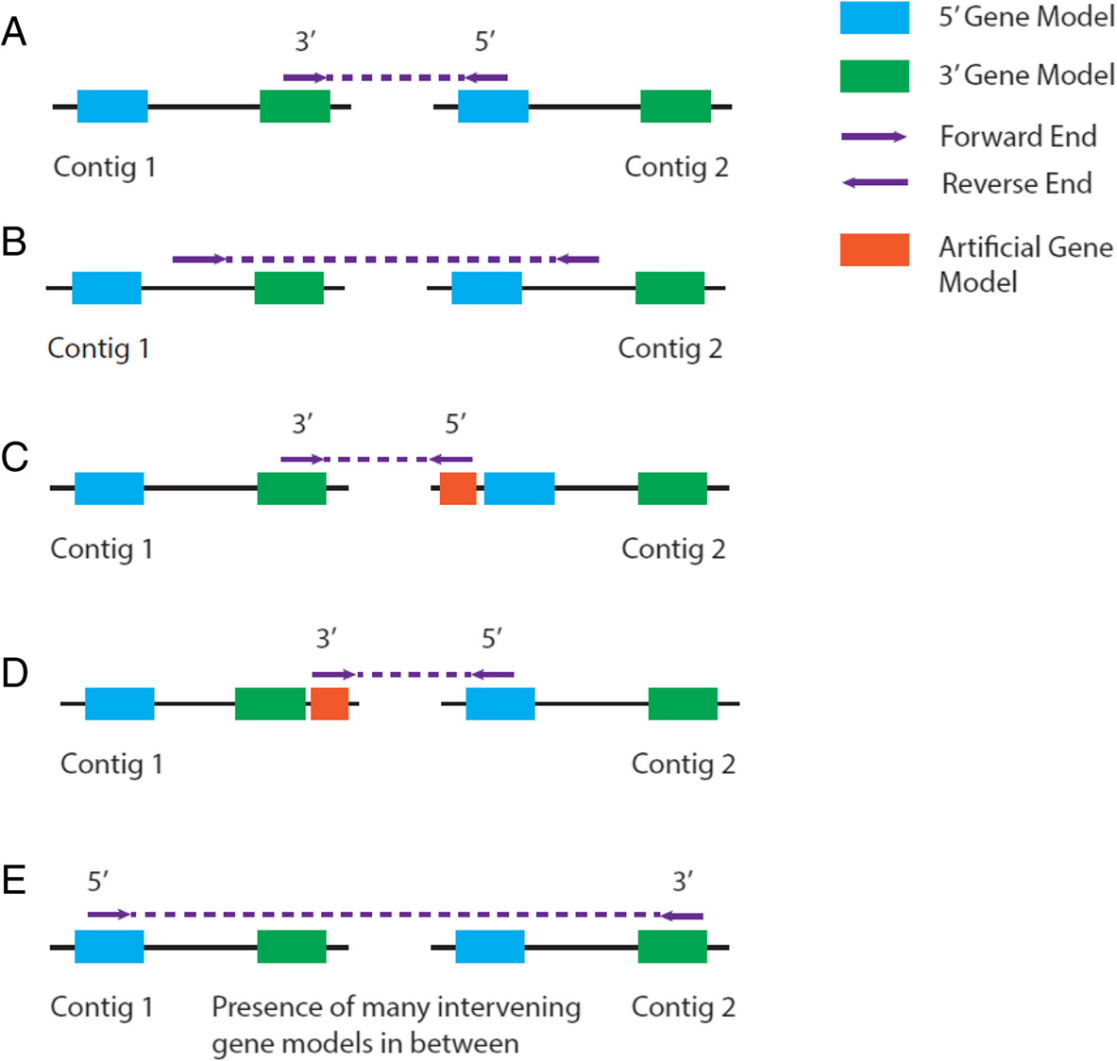
Denoise joining-pairs by first making sure they are mapped to 5′-most and 3′-most gene models. **a** For each joining-pair connecting two contigs, AGOUTI assigns each end (i.e., forward and reverse) to 5′-most and 3′-most gene models on the two contigs. In this case, the ends of the joined contigs have been labeled 3′ and 5′, respectively. Doing so ensures that each joining-pair does not span any gene models (i.e., there are no intervening gene models). **b** A joining-pair fails to map to any gene model at the edges of the two contigs. AGOUTI does not use such joining-pairs in scaffolding. **c** The reverse end of the joining-pair is mapped to 5′ of the 5′-most gene model on Contig 2. AGOUTI will create an artificial gene model accordingly, and assign an end label of 5′. **d** Similarly to (C), the forward end is mapped to 3′ of the 3′-most gene model on Contig 1. AGOUTI will create an artificial gene model and assign an end label of 3′. **e** Orientation imposes an important constraint. In this case, joining the contigs in the correct orientation shows that there are multiple intervening gene models between them, and this pair is therefore ignored. Here we only show the gene models at the edges of the contigs. There can be many genes in between them

Prior to scaffolding, AGOUTI denoises the joining-pairs by identifying and removing erroneous ones. Such pairs can result from many types of error, for example, from highly similar sequences on different chromosomes. The details of this denoising module are as follows. Because each read-pair comes from a single cDNA fragment, AGOUTI requires that it should not be separated by any number of genes in-between. This can be established by first checking whether the joining-pairs are mapped to the gene models at the edges of the contigs, that is, at 5′ and 3′. Specifically, AGOUTI labels each end of a joining-pair (i.e., left or right end) as 5 or 3 if it overlaps with the gene model at 5′ or 3′ of each contig (Fig. 2a). Each joining-pair is thus labeled either 5-3, 5-5, 3-5 or 3-3. If contigs contain only a single gene, reads overlapping the gene can be labeled either 5 or 3. It is worth noting that there are cases where the mapping positions of reads fail to overlap with gene models at either 5′ or 3′ ends. If joining-pairs fall between the terminal gene models in this way, they are excluded, as they are probably the result of highly similar sequences of genes in different parts of the genome (Fig. 2b). Otherwise, AGOUTI will retain the links and create artificial gene models at the corresponding locations (Fig. 2c, d). The artificial gene models not used in the scaffolding are discarded from the final updated gene annotation.

**Fig. 3:**
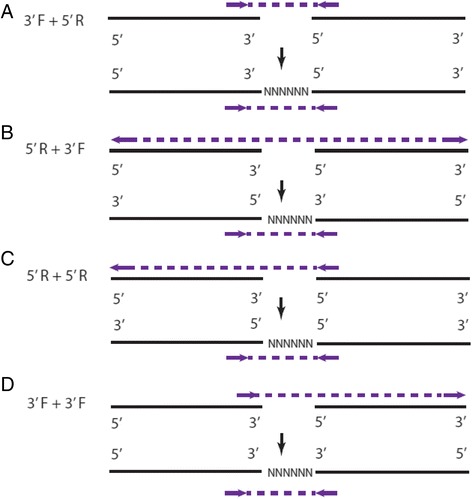
Denoise joining-pairs by further considering end-assignments with orientation constraints. The *top row* of each case shows the combination of the end-labels and orientation of a joining-pair. The *bottom row* demonstrates the orientation of the two contigs with the joining-pair after scaffolding. Because of the way each read-pair is sequenced (i.e., facing each other), we must ensure that the two contigs are scaffolded in a way such that this expected orientation is not violated. There are four combinations (**a**–**d** of the end-assignments and the orientation satisfying these requirements. For example, 5′R + 3′F means that one end of the joining-pair is mapped to the 5′-most gene model in the reverse orientation, while the other end is mapped to the 3′-most gene model in the forward orientation. If we reverse both sequences, we can make a valid scaffold between the two contigs using the joining-pair

In addition, to ensure that joining-pairs map to the edges of contigs, AGOUTI checks the orientation of the reads in these pairs to denoise the graph to be traversed. As both ends of a read-pair are inwardly sequenced, orientation imposes another important constraint and it must be considered in combination with the end assignments. For example, a joining-pair with a label of 5-3 and mapped in a forward-reverse fashion could span multiple intervening gene models, and should be removed (Fig. 2e). AGOUTI considers a pair of contigs for scaffolding as long as the joining-pairs supporting them follow one of the four valid combinations of end assignment and orientation, as demonstrated in Fig. 3a–d. AGOUTI also keeps track of the identities of the pair of gene models used to connect each contig pair, and their corresponding orientations.

### Scaffolding

**Fig. 4:**
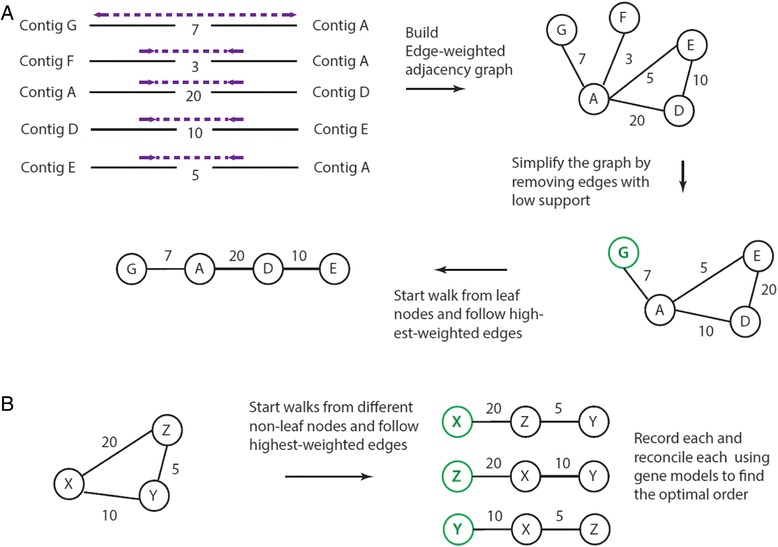
Scaffolding. **a** AGOUTI first builds an edge-weighted adjacency graph made up of contigs (*vertices; black lines*) and the joining-pairs between them (*edges; purple arrows*). Edges are weighted by the number of supporting joining-pairs. The graph is further simplified by removing edges with weight less than a user-specified value, and denoised using constraints described in the text and shown in Figs. 2 and 3. AGOUTI starts from leaf nodes (*green vertices*) and follows the highest-weighted edges. Each walk gives a scaffolding path, where the shortest such path has only two contigs. **b** Subgraphs with only non-leaf nodes that are ignored by RNAPATH. AGOUTI tries to traverse the subgraph starting from different vertices (shown in *green*). It records all the possible orders, each of which will be reconciled using constituent gene models to find the optimal one

AGOUTI carries out scaffolding by first building an edge-weighted adjacency graph using these joining-pairs (Fig. 4a). In the graph, each vertex represents a contig, and an edge connects two nodes if there are supporting joining-pairs between them. A weight is assigned to each edge according to the number of supporting joining-pairs. The graph is simplified by only keeping edges with a minimum weight (by default, K = 5).

AGOUTI traverses the graph from leaf nodes (i.e., those that connect to only one other contig), and follows the highest-weighted edges until no further extension can be made (Fig. 4a). For an edge to be traversed, it is required to have a minimum number of supporting joining-pairs, but AGOUTI makes this parameter (K) accessible from the command line. Each walk gives a scaffolding path, where the shortest such path includes only two contigs. This is the basic scaffolding procedure design in RNAPATH [5]. The RNAPATH scaffolding algorithm, however, ignores subgraphs made of only non-leaf vertices (Fig. 4b). Rather than randomly picking one, AGOUTI traverses such a subgraph from each of its nodes, following the highest-weighted edges. For the same group of vertices, AGOUTI records all possible traversal orders. AGOUTI will then identify a best order among them using the following steps.

**Fig. 5:**
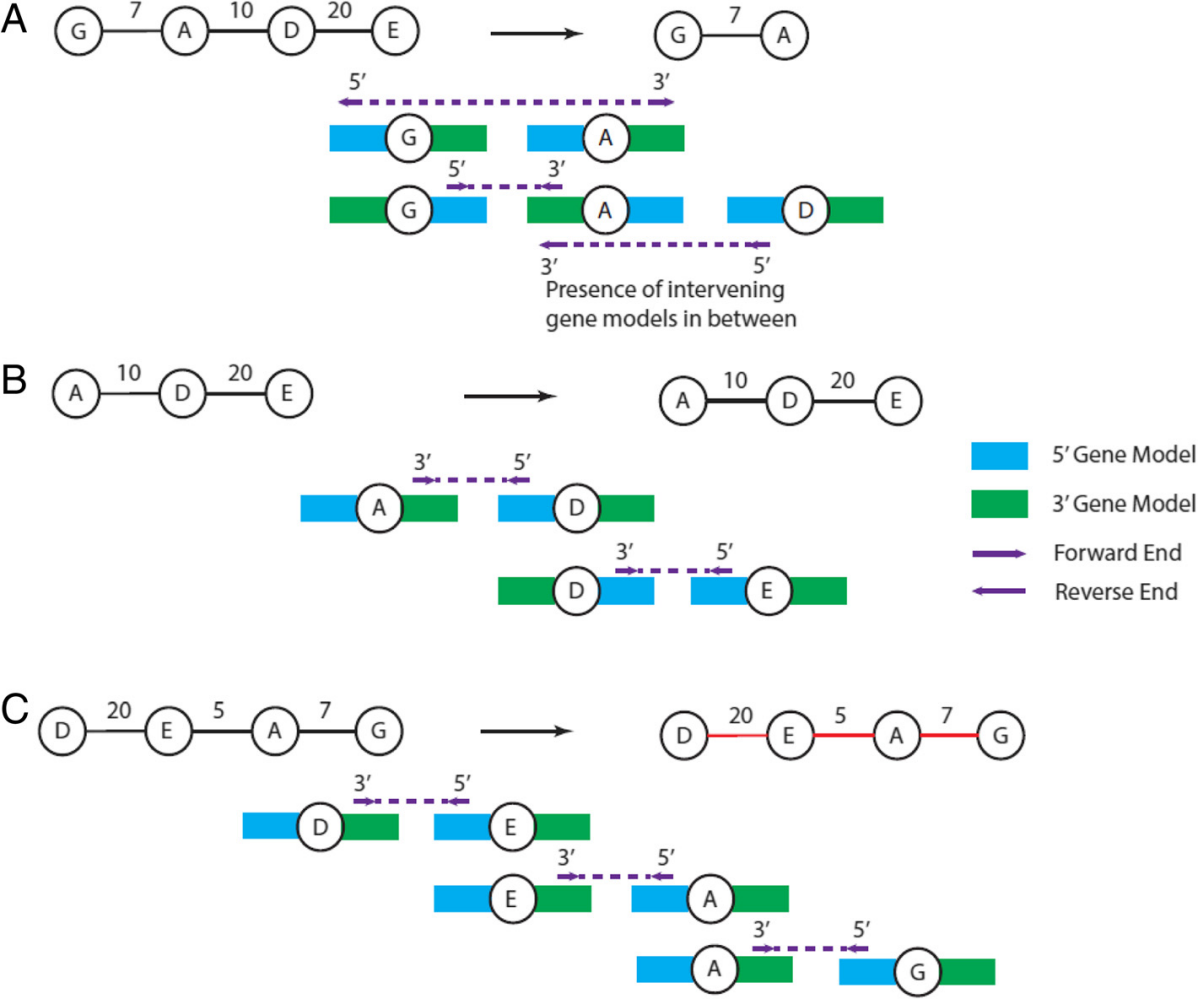
Scaffolding path reconciliation using constituent gene models. Each contig is denoted by a letter in a circle. The *blue* and *green* boxes represent the gene models at the 5′ and 3′ ends of a contig. Joining-pairs connecting two contigs are shown in purple, and orientation is indicated by arrows. Contigs are reverse-complemented as needed. **a** The scaffolding path obtained by following highest-weighted edges. Examining the gene model between each pair of the contigs in the path tells us that the extension from A to D violates the requirement for zero intervening gene models between two contigs. Therefore, the reconciled path contains only two contigs, rather than four. **b** The current best path is not the optimal one because it incorporates only a subset of all vertices. AGOUTI therefore picks another vertex and re-walks the subgraph. After reconciliation, the new path becomes the best path as it has more vertices than the previous one. **c** Similarly, AGOUTI next starts from node D and establishes a new path. The reconciled path contains all four vertices in the subgraph, and therefore AGOUTI uses it as the optimal one (edges shown in *red*) and stops checking other possible paths

For all the scaffolding paths, AGOUTI reconciles each one using constraints imposed by the constituent gene models. Specifically, it examines each pair of vertices in a path using the gene model making the connection (Fig. 5). This process terminates at any vertex whose connection with the next would have intervening gene models between them (Fig. 5). An optimal path is the one incorporating all of its vertices. AGOUTI will give up checking other possible paths once an optimal path is achieved. Otherwise, it will pick a different node, re-walk the subgraph, and reconcile the new path. After trying every vertex, AGOUTI will choose the path with the largest number of nodes. If there are two paths of equal length, AGOUTI will pick the path with the highest total weight; if there are two paths with equal weight, AGOUTI picks the first one in the list. AGOUTI marks all the vertices in the best path as visited and prevents them from being placed multiple times. In selecting an optimal scaffolding path, the reconciliation step prefers the smallest number of vertices over the highest total weight. This preference was established in response to observations that paths with the highest weights can have many connections, resulting in the presence of intervening gene models. In the future, it may be possible to extend the current greedy algorithm to a global optimal one with a score function of both weights and penalties on, for example, the number of intervening gene models.

Both the denoising and reconciliation steps check for intervening gene models between pairs of contigs, but in different contexts. The former checks for each pair alone, while the latter makes sure the condition still holds when multiple contigs within a scaffolding path are considered simultaneously (Fig. 5). The following provides an example of when both steps are needed. Consider the case of a three-exon gene spanning three contigs, A, B and C, where their true order is A→B→C. The denoising step makes sure that zero intervening gene models connect AB, AC and BC. We further denote the number of supports for AB, AC and BC as dAB, dAC and dBC, respectively. In cases where dAB < dAC < dBC, by following the highest weights the scaffolding algorithm indicates an order A→C→B, in which B and C are reversed. This reversal can spawn many intervening gene models between A and B, and/or B and C. The reconciliation step, therefore, serves as a reordering step, and can prevent AGOUTI from making intra-chromosomal errors (see evaluation below).

### Update

For each reconciled path, AGOUTI joins contigs into scaffolds, separating them by a gap of length defined by the user (1 kbp by default). Contigs are reverse-complemented whenever needed. AGOUTI also updates gene models according to the new assembly. For each pair of contigs within a scaffold, AGOUTI merges the two gene models from which the connection was made. The gene merge combines exons and converts coordinates to the new scaffold system. If contigs are reverse-complemented, all gene models on that contig will be reversed accordingly in the output annotation.

### AGOUTI applied to simulated assemblies

**Fig. 6:**
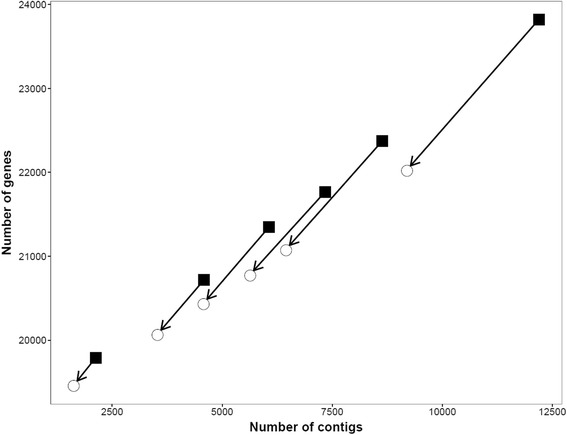
Performance of AGOUTI scaffolding with K = 5. AGOUTI was able to scaffold hundreds to thousands of contigs, and significantly reduced the number of gene models. The least contiguous assembly has the largest number of contigs scaffolded and the largest reduction in the number of gene models. The leftmost trace represents the most contiguous assembly, and the assembly represented on the far right is the least contiguous. The number of gene models is on the y-axis. The black squares and open circles indicate the number of gene models before and after the scaffolding, respectively

To evaluate the performance of AGOUTI (v0.3.2), we randomly fragmented the genome of the N2 strain of *Caenorhabditis elegans* ([12], version WS246) into six assemblies with varying numbers of contigs (CE1-CE6, Table 1). For each fragmented assembly, we performed gene prediction using AUGUSTUS (v 3.0.2) by setting ‘species = elegans’ and ‘gff = on’ [13]. We found that assemblies with larger numbers of contigs had increased numbers of predicted gene models (black squares in Fig. 6), consistent with results previously reported [2]. We used a single RNA-seq dataset from the same strain of *C. elegans* at the early embryo stage, obtained from modENCODE ([14], SRR316753, SRR317082 and SRR350977). We mapped these reads against each of our fragmented assemblies using BWA-MEM (v 0.7.10) with default settings [10], and used the mapping results [15], along with the predicted gene models, as inputs to AGOUTI. AGOUTI accepts results from any short-read mapper as long as: (1) it produces joining-pairs; (2) the results are in SAM/BAM format.

**Table 1:** Summary of six simulated genome assemblies and annotation

Assembly	No. of contigs	No. of predicted gene models
CE1	12,196	23,822
CE2	8,636	22,372
CE3	7,336	21,768
CE4	6,066	21,348
CE5	4,586	20,719
CE6	2,126	19,791
N2/CB	6,623	24,220
Lyco	103,352	85,058

### Evaluation of genome scaffolding

We evaluated the performance of AGOUTI on the six assemblies with both K = 5 and K = 2. AGOUTI was able to scaffold hundreds or thousands of contigs (open circles in Fig. 6), yielding higher scaffold N50 values (Table 2 and Additional file 1: Table S1). The most fragmented assembly had the largest number of contigs joined into scaffolds and the largest reduction in the number of gene models (Fig. 6). We checked the accuracy of contigs placed within each scaffold by comparing the output of AGOUTI with the N2 reference assembly. Across our simulated assemblies (CE1-CE6), AGOUTI achieved high accuracy by putting at least 99.98 % of contig pairs in the correct order (K = 2, Table 3). We found only a few pairs of contigs across our six assemblies that were incorrectly ordered (Intra-chromosomal error, Table 3), and a small number of cases where two contigs from different chromosomes were placed together (Inter-chromosomal error, Table 3).

**Table 2:** Summary of scaffolding performance of AGOUTI and RNAPATH with K = 2

Assembly	Program	No. of contigs scaffolded	No. of scaffolds in final assembly	Scaffold N50	No. of gene models in final assembly
CE1	AGOUTI	**5,349**	**8,525**	36,096	**21,776**
	RNAPATH	3,421	9,841	28,769	-
	RNAPATH^D^	5,323	8,528	**36,235**	-
CE2	AGOUTI	**3,879**	**5,974**	**73,881**	**20,951**
	RNAPATH	2,430	6,933	58,959	-
	RNAPATH^D^	3,869	5,976	73,770	-
CE3	AGOUTI	**3,093**	**5,243**	97,924	**20,658**
	RNAPATH	1,980	5,968	85,802	-
	RNAPATH^D^	3,082	5,244	**100,700**	-
CE4	AGOUTI	**2,678**	**4,242**	**127,283**	**20,324**
	RNAPATH	1,618	4,937	103,844	-
	RNAPATH^D^	2,671	**4,242**	**127,283**	-
CE5	AGOUTI	**1,966**	**3,284**	**258,507**	**19,978**
	RNAPATH	1,225	3,760	202,360	-
	RNAPATH^D^	1,961	3,285	**258,507**	-
CE6	AGOUTI	**941**	**1,501**	**642,283**	**19,411**
	RNAPATH	511	1,774	492,192	-
	RNAPATH^D^	934	1,504	**642,283**	-
	AGOUTI	1,358	5,743	73,952	**23,666**
N2/CB	RNAPATH	762	6,118	65,196	-
	RNAPATH^D^	**1,376**	**5,722**	**74,347**	-
	AGOUTI	**20,594**	89,452	27,524	**79,222**
Lyco	RNAPATH	8,797	97,181	24,127	-
	RNAPATH^D^	20,529	**89,301**	**28,202**	-

^D^RNAPATH run with denoised joining-pairs. Best-performing programs are highlighted in bold

**Table 3:** Scaffolding accuracy of AGOUTI and RNAPATH with K = 2

Assembly	Program	Inter-chromosomal errors	Intra-chromosomal errors	No. of contigs placed repeatedly
CE1	AGOUTI	**2**	**2**	**0**
	RNAPATH	6	7	12
	RNAPATH^D^	8	10	**0**
CE2	AGOUTI	**2**	**0**	**0**
	RNAPATH	8	14	1
	RNAPATH^D^	3	12	**0**
CE3	AGOUTI	**0**	**1**	**0**
	RNAPATH	3	10	11
	RNAPATH^D^	2	11	**0**
CE4	AGOUTI	**1**	**0**	**0**
	RNAPATH	7	5	10
	RNAPATH^D^	2	7	**0**
CE5	AGOUTI	**0**	**0**	**0**
	RNAPATH	1	2	**0**
	RNAPATH^D^	1	4	**0**
CE6	AGOUTI	**1**	**0**	**0**
	RNAPATH	6	4	**0**
	RNAPATH^D^	**1**	3	**0**
	AGOUTI	**17**	**3**	**0**
N2/CB	RNAPATH	37	14	6
	RNAPATH^D^	20	12	**0**
	AGOUTI	**213**	**12**	**0**
Lyco	RNAPATH	535	150	17
	RNAPATH^D^	366	292	**0**

^D^RNAPATH run with denoised joining-pairs. Best-performing programs are highlighted in bold

### Comparison of AGOUTI and RNAPATH

We compared results using AGOUTI with results obtained from RNAPATH, across a range of different input values. To our knowledge, RNAPATH is the only program that uses RNA-seq without further transcriptome assembly (e.g., [5]) to scaffold genomes. Across all conditions, AGOUTI found more connections than RNAPATH (Table 2 and Additional file 1: Table S1) and produced fewer errors (Table 3 and Additional file 1: Table S2).

One major difference between AGOUTI and RNAPATH is the denoising step AGOUTI performs prior to scaffolding, which removes erroneous joining-pairs. We expected a noise-free graph to result in better scaffolding. We tested this by running RNAPATH on the same six assemblies on which AGOUTI was tested. More specifically, we compared the performance of these algorithms on two datasets, one with all the joining-pairs (including noisy pairs), and the other using only the noise-free ones. Both sets of joining-pairs came from the same RNA-seq data. We also used the default settings of RNAPATH (i.e., K = 2) for both tests. Consistent with our expectation, RNAPATH, with the additional noisy edges, recovered fewer contigs across all six assemblies (Table 2). This number was boosted when the noise-free data was used (compare RNAPATH with RNAPATH^D^ in Table 2).

Second, the scaffolding algorithm in AGOUTI is guided by evidence from gene models, in addition to weights. We expected this to result in more accurate scaffolding even when noise-free datasets were used. On the basis of the runs on the noise-free datasets described above, we found that RNAPATH suffered from many more inter-chromosomal errors than AGOUTI (Table 3). These errors occurred as a result of joining contigs from different chromosomes. In addition, RNAPATH produced intra-chromosomal errors that placed contigs of the same chromosome in the wrong order. We also observed that RNAPATH repeatedly incorporated the same contigs into different scaffolds when given noisy data, but these errors disappeared with the denoised read-pairs (compare RNAPATH with RNAPATH^D^ in Table 3).

These differences in error rate could be due to the difference in the minimum number of joining-pairs required by AGOUTI and RNAPATH, rather than the scaffolding algorithms themselves. We tested this by re-running RNAPATH on the six noise-free datasets, and increasing the minimum number of supporting joining-pairs to 5 (i.e., K = 5). With this larger number, RNAPATH still generated more error-prone results than AGOUTI (Additional file 1: Table S2).

Finally, there were paths scaffolded by AGOUTI that were entirely missed by RNAPATH, for example, a path consisting of only non-leaf vertices (Fig. 4b). Because RNAPATH initiates a graph walk only from leaf nodes (and these have outdegree = 1) it ignores paths without leaves. In a comparison of the results from AGOUTI and RNAPATH, the former always placed more contigs regardless of parameter settings.

### Evaluation of genome annotation

**Fig. 7:**
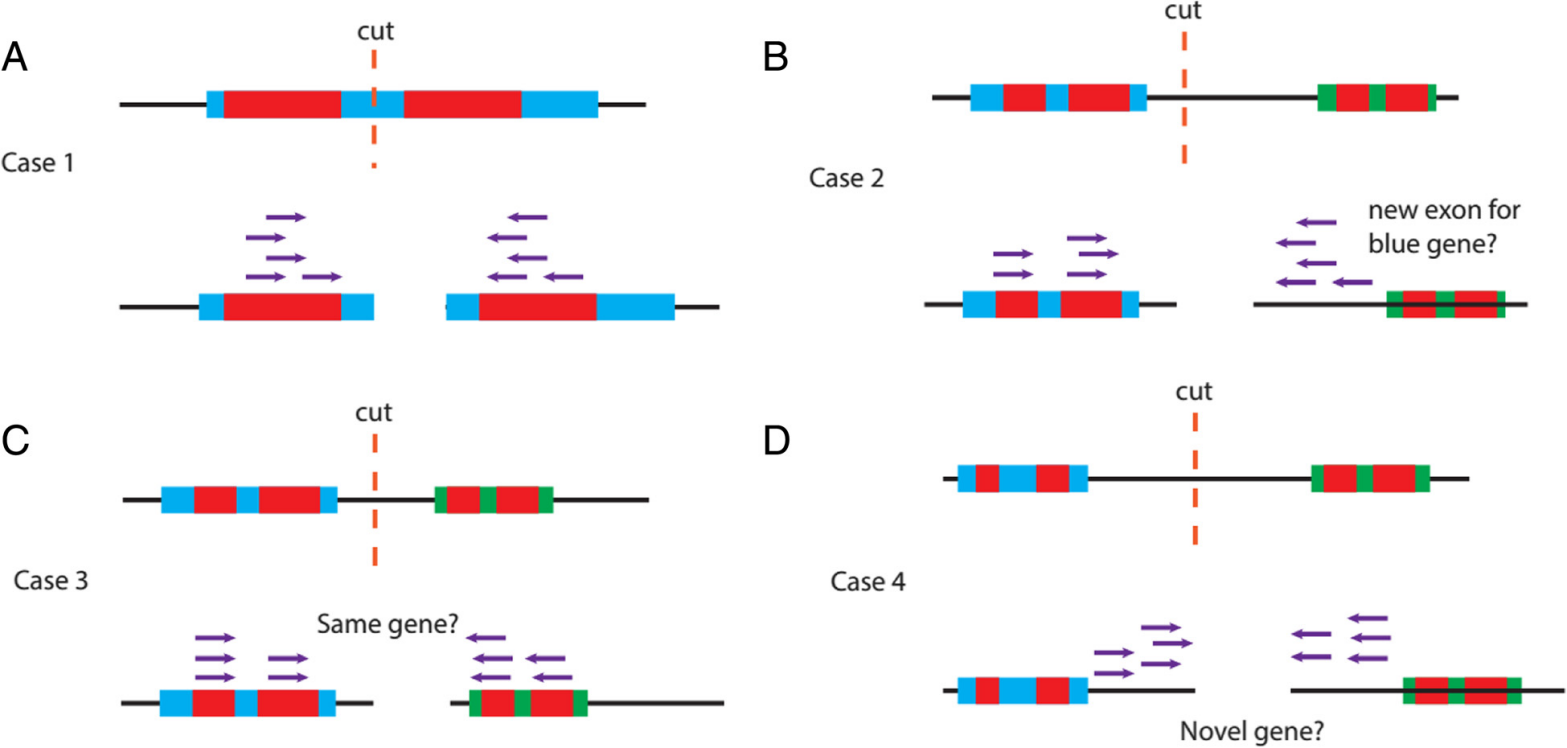
Evaluation of whether each pair of contigs was connected because of the existence of an underlying gene. The *top* row of each panel shows a single sequence that will be assembled into two contigs. The site where the split in the assembly occurs is indicated by ‘cut’. The *bottom* row of each panel shows, for the two contigs, whether they are brought together because of exons of the same gene. Blue and green boxes represent genes, with the red boxes inside them representing exons; arrows in purple represent joining-pairs of reads. **a** Case 1. Two contigs are connected by AGOUTI because they carry exons of the same gene. Approximately 95 % of the contig pairs scaffolded by AGOUTI fell into this category. **b** Case 2. Only one end of a joining-pair overlaps a predicted gene on either contig. This suggests the existence of a new exon in one of the predicted genes. **c** Case 3. The joining-pairs are mapped to two different annotated genes in the *C. elegans* genome. This suggests that the two genes should be merged into one, or that there was a failure of transcriptional termination such that reads connected two adjacent genes. **d** Case 4. The joining-pairs are not mapped to any predicted genes, which may indicate the existence of a novel gene

We also investigated whether the connections between contigs made by AGOUTI accurately reflected the existence of underlying genes. Specifically, we asked of each contig pair whether the joining-pairs used for scaffolding were mapped to two exons of a single gene (Fig. 7). We used the gene annotation of the same version as the reference N2 genome to evaluate these connections [12]. Within each assembly, approximately 95 % of genes joined by AGOUTI connected two exons of the same annotated gene (using a minimum of five joining-pairs; Case 1, Fig. 7a; Table 4). Among the rest of the contig pairs, some connected an exon on one contig with an unannotated exon on the other contig (Case 2, Fig. 7b; 2 % of cases). Another class of genes merged by joining-pairs had mappings to two different genes (Case 3, Fig. 7c; 2 %). This suggests that the two genes should be merged into one, or that there was a failure of transcriptional termination such that reads connect two adjacent genes. In a final scenario, both ends of the joining-pairs failed to map to any known genes on either contig, suggesting a potential novel gene (Case 3, Fig. 7d; 1 %). For consecutive pairs of contigs (i.e., pairs that are physically next to each other on a chromosome), we considered these notable cases to be a bonus feature of AGOUTI and did not count them as false positives; the number of each type is listed in Table 4.

**Table 4:** Evaluation of AGOUTI scaffolding in terms of gene models

		Number of contig pairs scaffolded				
Assembly	K	Total	Case 1	Case 2	Case 3	Case 4
CE1	2	3,671	3,427	92 (74)^a^	75 (57)	77 (67)
	5	2,994	2,858	56 (40)	56 (40)	24 (21)
CE2	2	2,660	2,463	72 (55)	47 (34)	78 (68)
	5	2,184	2,072	39 (29)	39 (26)	34 (27)
CE3	2	2,093	1,928	59 (44)	42 (33)	64 (60)
	5	1,699	1,611	36 (24)	25 (20)	27 (25)
CE4	2	1,822	1,697	50 (39)	32 (26)	43 (38)
	5	1,486	1,424	24 (16)	26 (21)	12 (10)
CE5	2	1,302	1,215	41 (34)	20 (18)	26 (21)
	5	1,054	1,011	18 (13)	13 (13)	12 (8)
CE6	2	624	582	22 (19)	6 (5)	14 (10)
	5	501	483	10 (7)	4 (4)	4 (1)

^a^The figures in parentheses show the number of consecutive contig pairs in each case

### AGOUTI applied to additional assemblies

We tested AGOUTI under two additional scenarios. First, we sequenced a highly heterozygous, outbred individual of *C. elegans* that was the result of a cross between the N2 and CB4856 strains, with 50X fragment libraries and 45X mate-pair libraries. We built an initial genome assembly with ALLPATHS-LG using all default settings ([16], release 51646). We evaluated AGOUTI on this assembly in contig form (N2/CB, Table 1). Second, we chose the domesticated tomato, *S. lycopersicum*, which represents a test of AGOUTI on a larger and more complex genome [17]. We downloaded its genome (v2.50) from the SOL Genomics Network, and randomly split it in a similar fashion as we did with the simulated *C. elegans* assemblies (Lyco, Table 1). We obtained RNA-seq reads for *S. lycopersicum* from a recent study of 13 species of wild tomato [18]. We repeated the gene prediction with AUGUSTUS, and read-mapping using BWA-MEM, on both assemblies as described earlier.

We evaluated the performance of AGOUTI on the two assemblies with K = 2 and checked the accuracy by comparing the output of AGOUTI with the N2 reference. Consistently, AGOUTI was able to scaffold thousands of contigs and merge hundreds to thousands of fragmented gene models for both assemblies (Table 2). RNAPATH, however, struggled to join as many contigs with the noisy data. Its performance was boosted when noise-free joining-pairs were provided (Table 2). This result emphasizes the importance of denoising prior to scaffolding. In terms of accuracy, AGOUTI consistently committed fewer errors when compared with RNAPATH, with the obvious differences falling in the intra-chromosomal category (Table 3). This suggests that the heuristic of following the highest weights can lead to many incorrect paths, and our reconciliation is able to derive the true order by taking into account features of gene models.

We noticed that AGOUTI scaffolded fewer contigs for the real *C. elegans* assembly than the simulated ones. One possible explanation is that there are not as many breakpoints as in the simulated genome of the N2/CB assembly. The 24,000 predicted gene models, however, suggest otherwise (Table 1). We calculated and compared the percentage of breakpoints falling within the non-coding regions of the N2/CB, and all six simulated, assemblies. This was done by first finding coordinates of breakpoints on the N2 reference, and then examining overlaps with annotations of protein-coding genes using BEDTools [19]. We designated a breakpoint as intergenic if it did not intersect with any genic intervals. In total, we observed no excess of intergenic breakpoints in the N2/CB assembly compared to CE1-CE6 (41 % versus 38 %, 38 %, 39 %, 38 %, 41 % and 41 %, respectively). Another possibility is a difference in the number of joining-pairs found in each assembly. Given the same number of breakpoints, we expected that fewer joining-pairs would make fewer connections. We thus compared the numbers of joining-pairs found in the N2/CB and CE1-CE4 assemblies and observed an almost three-fold difference among them (202,264 versus 519,444, 382,836, 578,406 and 261,308, respectively). This is not surprising as we mapped RNA-seq reads sequenced from the N2 strain to the assembly carrying not only the N2 alleles but also the CB4856 ones. The sequence divergence between the two strains alone can prevent many reads from being mapped [20]. Lastly, heterozygous individuals pose great challenges for genome assemblers, and one such error is known as allelic splitting [2]. Allelic splitting refers to the case where alleles (haplotypes) at the same locus are incorrectly assembled as paralogous loci, thereby inflating the number of predicted gene models. It is highly likely that many of the 24,000 gene models predicted from the N2/CB assembly fell into this category. Because AGOUTI is not designed to fix gene models that result from allelic splitting, it makes sense that we have seen less of an impact.

### Running time and memory usage

We compared running time and maximum memory usage between AGOUTI and RNAPATH on the CE1-CE6, N2/CB and Lyco assemblies. All tests were done on an HP DL360 server with two Intel Xeon E5-2600 processors and 24 GB of RAM. We ran RNAPATH on noise-free datasets to enable fair comparisons. AGOUTI was at least 100 times faster than RNAPATH in constructing graphs and scaffolding, and consumed a low amount of memory (Table 5). These differences reached a maximum when the Lyco assembly was evaluated. In addition, we tested the running time of denoising and reconciliation, the steps that give AGOUTI an advantage over RNAPATH. Both modules ran very efficiently and finished within 2 min for the 750 Mbp tomato assembly (Table 5). This suggests that AGOUTI can be applied not only to species with smaller genomes, but also those with larger ones.

**Table 5:** Comparison of running times and maximum memory for AGOUTI and RNAPATH

Assembly	Programs	Graph building^a^	Scaffolding^a^	Denoising^a^	Reconciliation^a^	Max. memory^b^
CE1	AGOUTI	0.018	2.412	17.4	1.152	0.248
	RNAPATH^D^	6.342	130.2	-	-	0.421
CE2	AGOUTI	0.012	0.342	7.2	0.882	0.23
	RNAPATH^D^	5.232	64.2	-	-	0.273
CE3	AGOUTI	0.012	0.27	15.6	0.66	0.267
	RNAPATH^D^	5.142	45.6	-	-	0.24
CE4	AGOUTI	0.018	0.21	5.4	0.588	0.22
	RNAPATH^D^	2.358	34.8	-	-	0.16
CE5	AGOUTI	0.012	0.132	3.6	0.39	0.202
	RNAPATH^D^	1.278	16.8	-	-	0.161
CE6	AGOUTI	0.0006	0.06	3	0.192	0.204
	RNAPATH^D^	0.762	3.6	-	-	0.172
N2/CB	AGOUTI	0.012	0.078	2.4	0.288	0.188
	RNAPATH^D^	0.912	17.4	-	-	0.205
Lyco	AGOUTI	0.09	5.4	87	4.8	1.389
	RNAPATH^D^	374.148	7,186.8	-	-	8.994

^a^The numbers represent seconds. ^b^The numbers represent gigabytes

## Conclusions

AGOUTI is a powerful and effective scaffolder and, unlike most scaffolders, is expected to become more effective in larger genomes because of the commensurate increase in intron length. AGOUTI is able to scaffold thousands of contigs while simultaneously reducing the number of gene models by hundreds or thousands, making it easier to improve both genome assemblies and genome annotations.

## Availability and requirements

**• Project name:** AGOUTI

**•Project home page:**https://github.com/svm-zhang/AGOUTI

**• Operating system(s):** Linux

**• Programming language:** Python

**• Requirements:** Python 2.7 or higher

**• License:** MIT

## Additional files

Additional file 1: Supplementary tables. Scaffolding performance and accuracy of AGOUTI and RNAPATH with K = 5. (DOCX 16 kb) Additional file 2: Supporting data description. (DOCX 14 kb)
