# Supplementary material for: AGOUTI: improving genome assembly and annotation using transcriptome data
Source: Gigascience. 2016 Jul 19;5:31. doi: 10.1186/s13742-016-0136-3 (PMC4952227; doi:10.1186/s13742-016-0136-3)
Supplement: Additional file 1: — Supplementary tables. Scaffolding performance and accuracy of AGOUTI and RNAPATH with K = 5. (DOCX 16 kb) [file 13742_2016_136_MOESM1_ESM.docx]

**Supplementary table 1. Summary of scaffolding performance of AGOUTI and RNAPATH with K = 5**

| Assembly | Program | No. of contigs scaffolded | No. of scaffolds in final assembly | Scaffold N50 | No. of gene models in final assembly |
| --- | --- | --- | --- | --- | --- |
| 1 | AGOUTI | **4,448** | **9,201** | **32,611** | **22,021** |
|  | RNAPTH^D^ | 4,412 | 9,222 | **32,611** | - |
|  |  |  |  |  |  |
| 2 | AGOUTI | **3,235** | **6,452** | **66,927** | **21,071** |
|  | RNAPATH^D^ | 3,205 | 6,469 | **66,927** | - |
|  |  |  |  |  |  |
| 3 | AGOUTI | **2,541** | **5,637** | **97,667** | **20,770** |
|  | RNAPATH^D^ | 2,528 | 5,642 | **97,667** | - |
|  |  |  |  |  |  |
| 4 | AGOUTI | **2,239** | **4,579** | **119,046** | **20,433** |
|  | RNAPATH^D^ | 2,232 | **4,579** | **119,046** | - |
|  |  |  |  |  |  |
| 5 | AGOUTI | **1,621** | **3,531** | **231,117** | **20,063** |
|  | RNAPATH^D^ | 1,608 | 3,539 | **231,117** | - |
|  |  |  |  |  |  |
| 6 | AGOUTI | **766** | **1,625** | **566,481** | **19,455** |
|  | RNAPATH^D^ | 763 | **1,625** | **566,481** | - |

^D^RNAPATH run with denoised joining-pairs. Best-performing programs are highlighted in bold.

**Supplementary table 2. Scaffolding accuracy of AGOUTI and RNAPATH with K = 5**

| Assembly | Program | Inter-chromosomal errors | Intra-chromosomal errors | No. of contigs placed repeatedly |
| --- | --- | --- | --- | --- |
| 1 | AGOUTI | **1** | **0** | **0** |
|  | RNAPATH^D^ | 2 | 4 | 0 |
|  |  |  |  |  |
| 2 | AGOUTI | **0** | **0** | **0** |
|  | RNAPATH^D^ | 1 | 4 | 0 |
|  |  |  |  |  |
| 3 | AGOUTI | **0** | **0** | **0** |
|  | RNAPATH^D^ | 0 | 4 | 0 |
|  |  |  |  |  |
| 4 | AGOUTI | **1** | **0** | **0** |
|  | RNAPATH^D^ | 1 | 9 | 0 |
|  |  |  |  |  |
| 5 | AGOUTI | **0** | **0** | **0** |
|  | RNAPATH^D^ | 0 | 1 | 0 |
|  |  |  |  |  |
| 6 | AGOUTI | **0** | **0** | **0** |
|  | RNAPATH^D^ | 0 | 2 | 0 |

^D^RNAPATH run with denoised joining-pairs. Best-performing programs are highlighted in bold.
