# Supplementary material for: AGOUTI: improving genome assembly and annotation using transcriptome data
Source: Gigascience. 2016 Jul 19;5:31. doi: 10.1186/s13742-016-0136-3 (PMC4952227; doi:10.1186/s13742-016-0136-3)
Supplement: Additional file 2: — Supporting data description. (DOCX 14 kb) [file 13742_2016_136_MOESM2_ESM.docx]

**AGOUTI: improving genome assembly and annotation using transcriptome data**

Simo V. Zhang, Luting Zhuo and Matthew W. Hahn

Abstract

**Background:** Genomes sequenced using short-read, next-generation sequencing technologies can have many errors and may be fragmented into thousands of small contigs. These incomplete and fragmented assemblies lead to errors in gene identification, such that single genes spread across multiple contigs are annotated as separate gene models. Such biases can confound inferences about the number and identity of genes within species, as well as gene gain and loss between species.

**Results:** We present AGOUTI (Annotated Genome Optimization Using Transcriptome Information), a tool that uses RNA-seq data to simultaneously combine contigs into scaffolds and fragmented gene models into single models. We show that AGOUTI improves both the contiguity of genome assemblies and the accuracy of gene annotation, providing updated versions of each as output. Running AGOUTI on both simulated and real datasets, we show that it is highly accurate and that it achieves higher accuracy and contiguity compared to other existing methods.

**Conclusion:** AGOUTI is a powerful and effective scaffolder, and unlike most scaffolders is expected to become more effective in larger genomes because of the commensurate increase in intron length. AGOUTI is able to scaffold thousands of contigs while simultaneously reducing the number of gene models by hundreds to thousands. The software is available free of charge under the MIT license from https://github.com/svm-zhang/AGOUTI.

**Data types:** genome assembly, RNA-seq, NGS reads mapping results, genome annotation, RNAPATH script used in experiments

**Organism:** *C. elegans*, RNA-seq extracted from early embryo stage, *S. lycopersicum*

**Dataset:** all simulated and real assemblies in FASTA, Fastq files of RNA-seq reads, BAM files of reads mapping, GFFs of genome annotation, and all AGOUTI results

**Dataset size:** 40 GB

**Dataset DOI:** http://dx.doi.org‎/10.5524/100195

**Readme file:** https://github.com/svm-zhang/AGOUTI
